# Supplementary material for: Pan-genome analysis of 13 Malus accessions reveals structural and sequence variations associated with fruit traits
Source: Nat Commun. 2023 Nov 15;14:7377. doi: 10.1038/s41467-023-43270-7 (PMC10651928; doi:10.1038/s41467-023-43270-7)
Supplement: Supplementary file 1 — Supplementary Information [file 41467_2023_43270_MOESM1_ESM.pdf]

Pan-genome analysis of 13 *Malus* accessions reveals structural and sequence variations associated with fruit traits

Wang *et al*

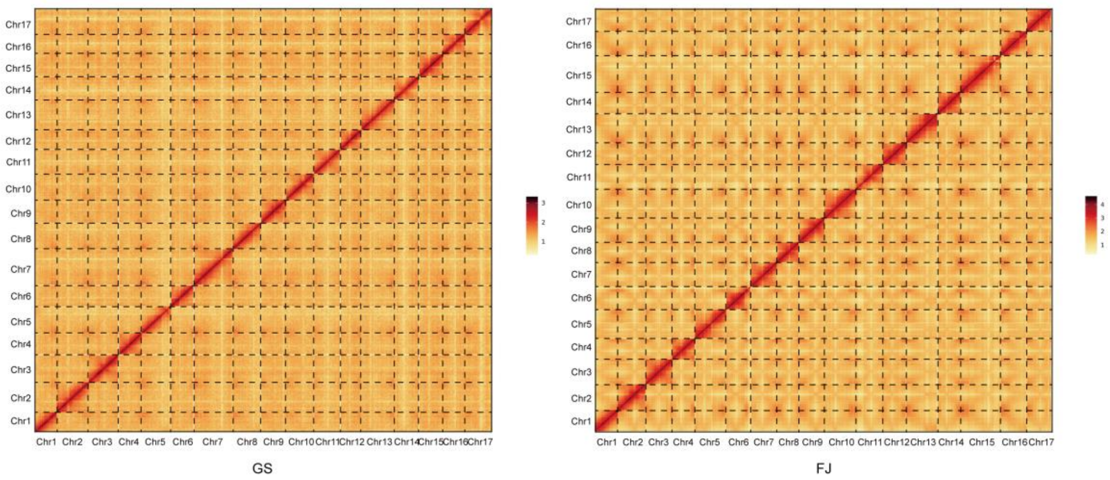

Supplementary Fig. 1. Genome-wide contact matrix of the GS and FJ genomes. The color intensity indicates the frequency of contact between two 100 kb loci. The map shows high-resolution individual superscaffolds, which were scaffolded and assembled independently.

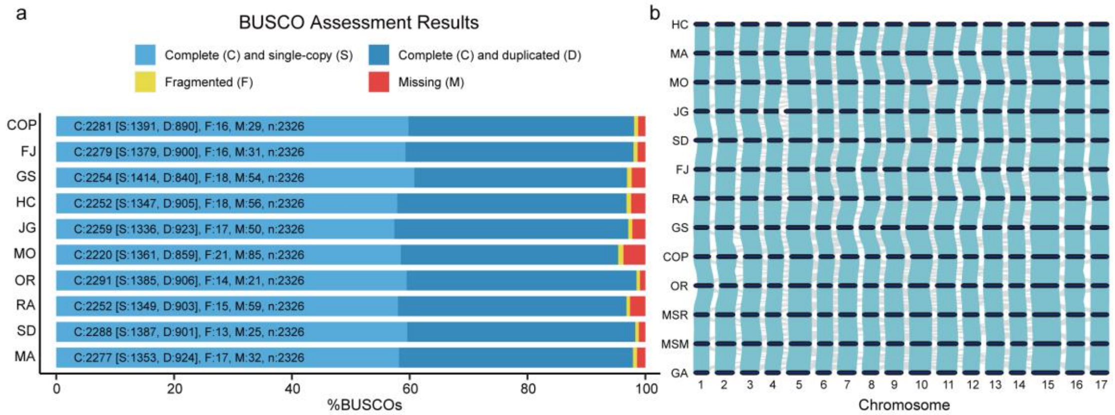

Supplementary Fig. 2. Genome assembly of ten apple accessions. a BUSCO evaluation of apple genome assemblies. Gene space coverage of apple genome assemblies was assessed using 2,326 core conserved plant genes with BUSCO (https://busco.ezlab.org/). **b** Genomic synteny between homologous chromosomes (haplomes) of the diploid assemblies were constructed by *MCSCanX*. Numbers indicate chromosomes.

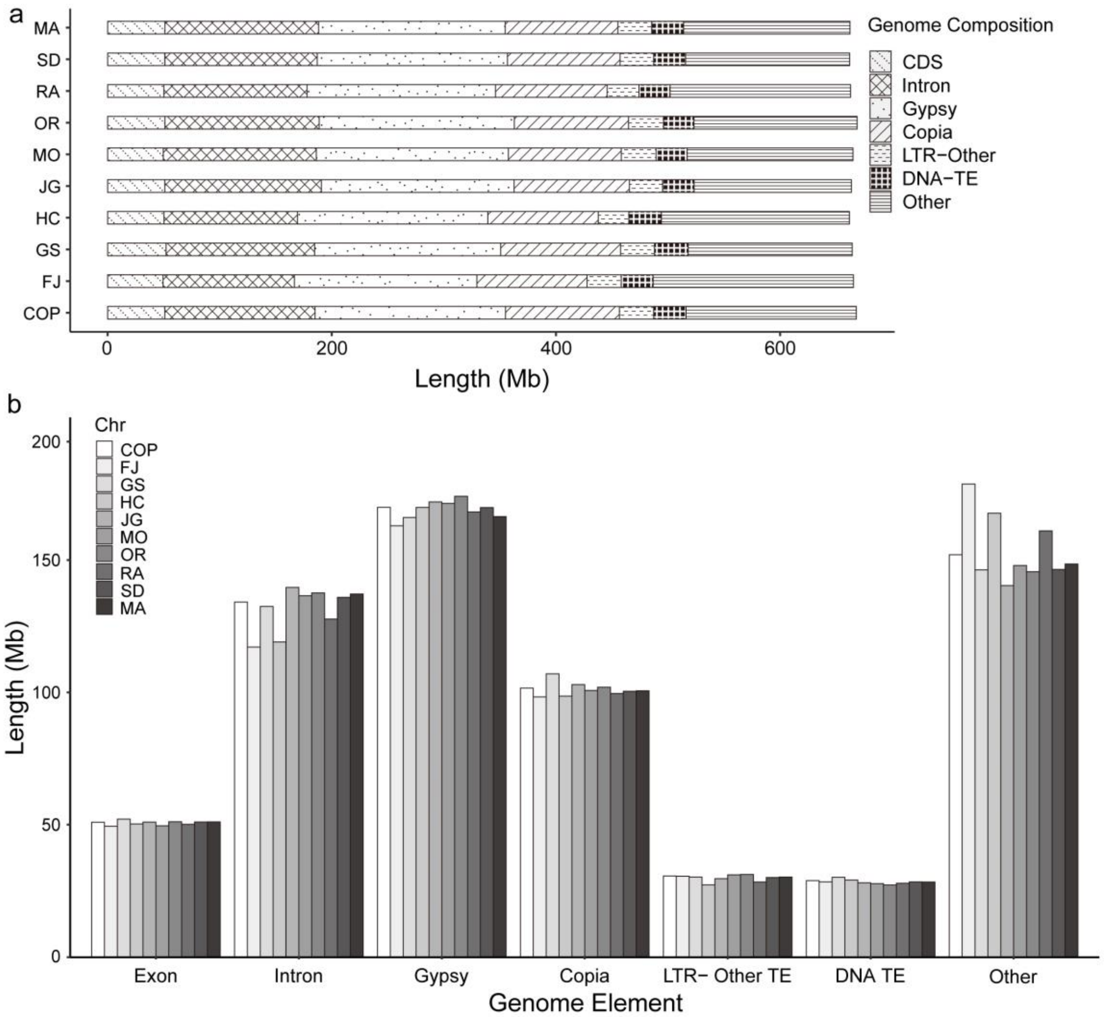

Supplementary Fig. 3. Genome composition of ten apple accessions. a Genome composition of the ten genomes. **b** Element composition in the ten genomes.

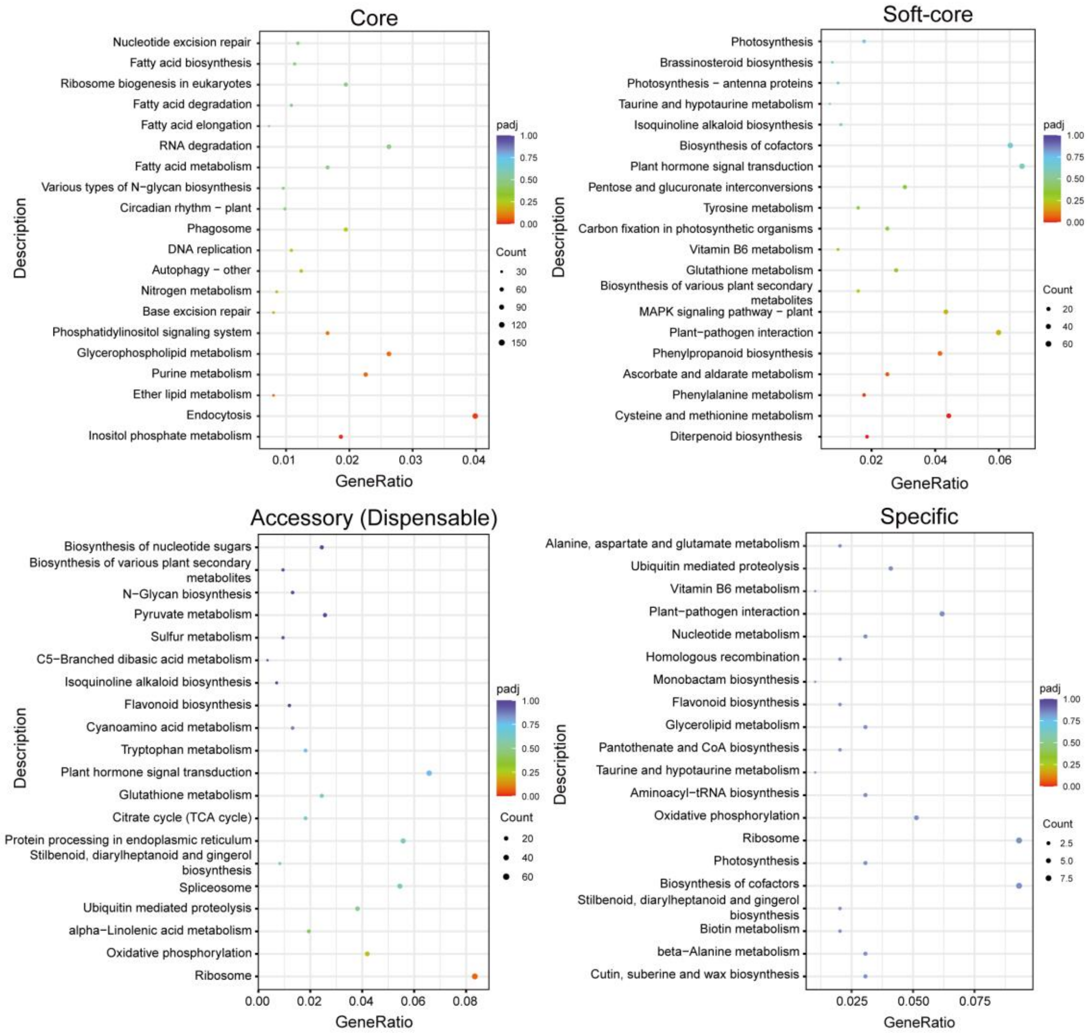

Supplementary Fig. 4. KEGG (Kyoto Encyclopaedia of Genes and Genomes) pathway enrichment analysis of core genes, soft-core genes, accessory (dispensable) genes and specific genes in the 13 apple accessions.

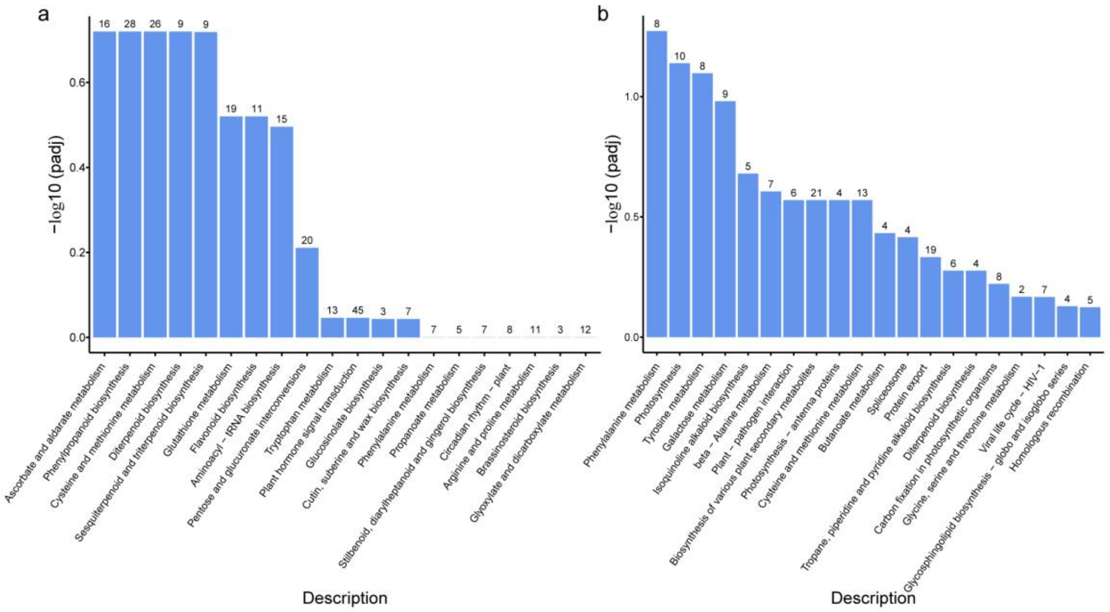

Supplementary Fig. 5. KEGG pathway enrichment analysis. a KEGG pathway enrichment analysis of genes only existed in four wild-type apples. **b** KEGG pathway enrichment analysis of the differentially expressed genes between colored and uncolored apple fruit and only high expressed genes in the colored apple of them were shown in the figure.

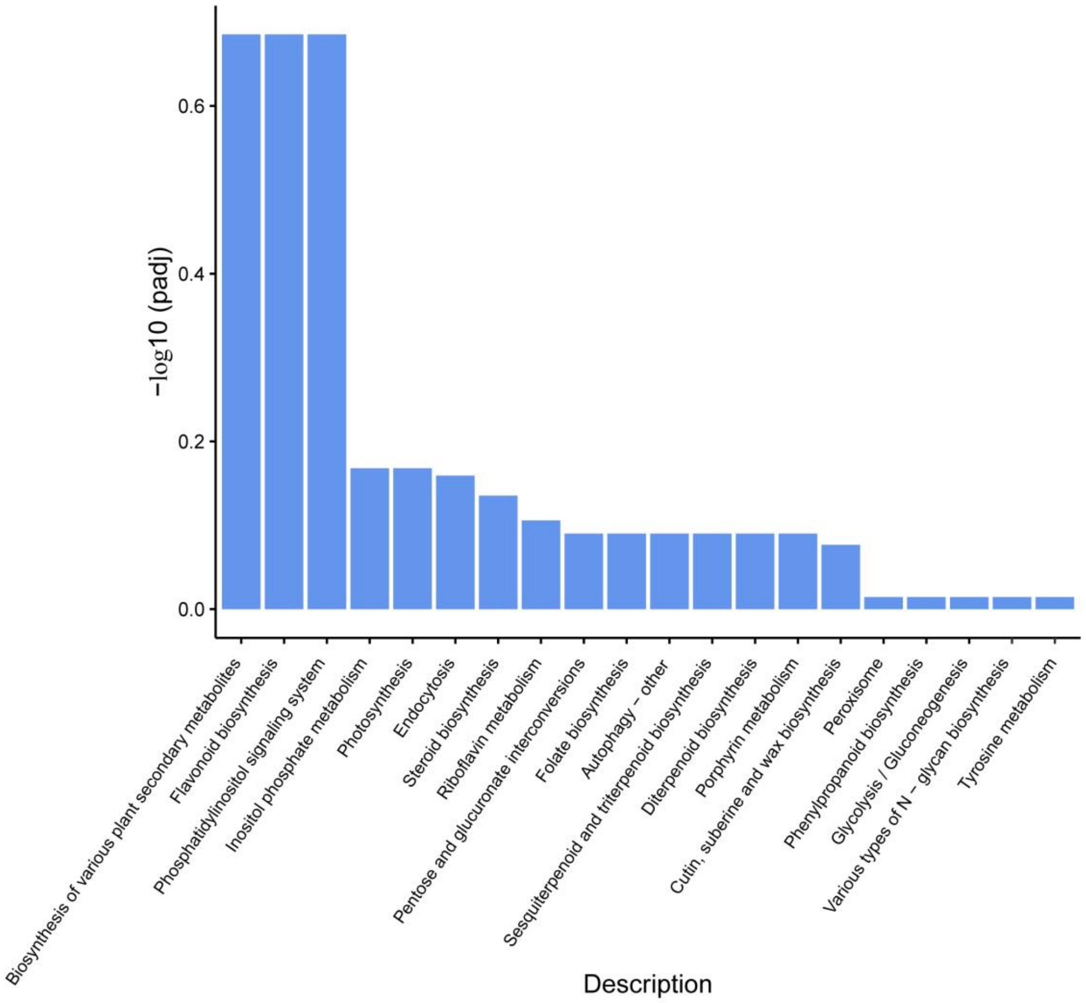

Supplementary Fig. 6. KEGG pathway enrichment analysis of the up-regulated genes in colored apple fruit compared with uncolored apple fruit.

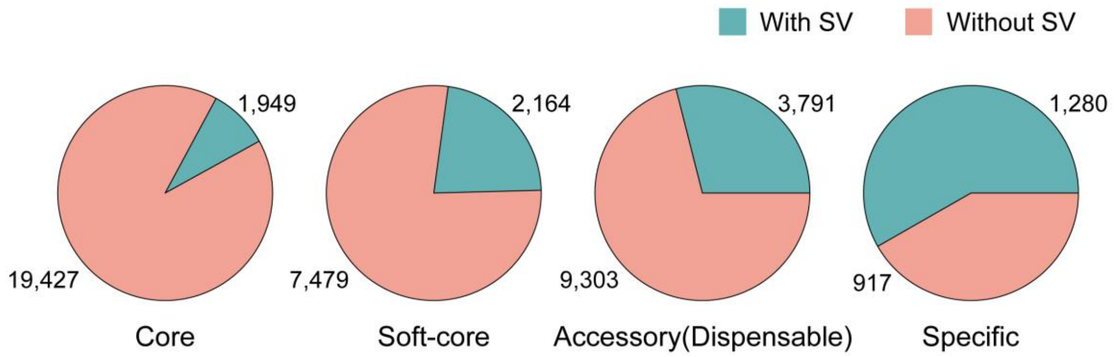

Supplementary Fig. 7. The cross-analysis of SVs and gene clusters. SVs overlapping of core, soft-core, accessory (dispensable) and specific genes.

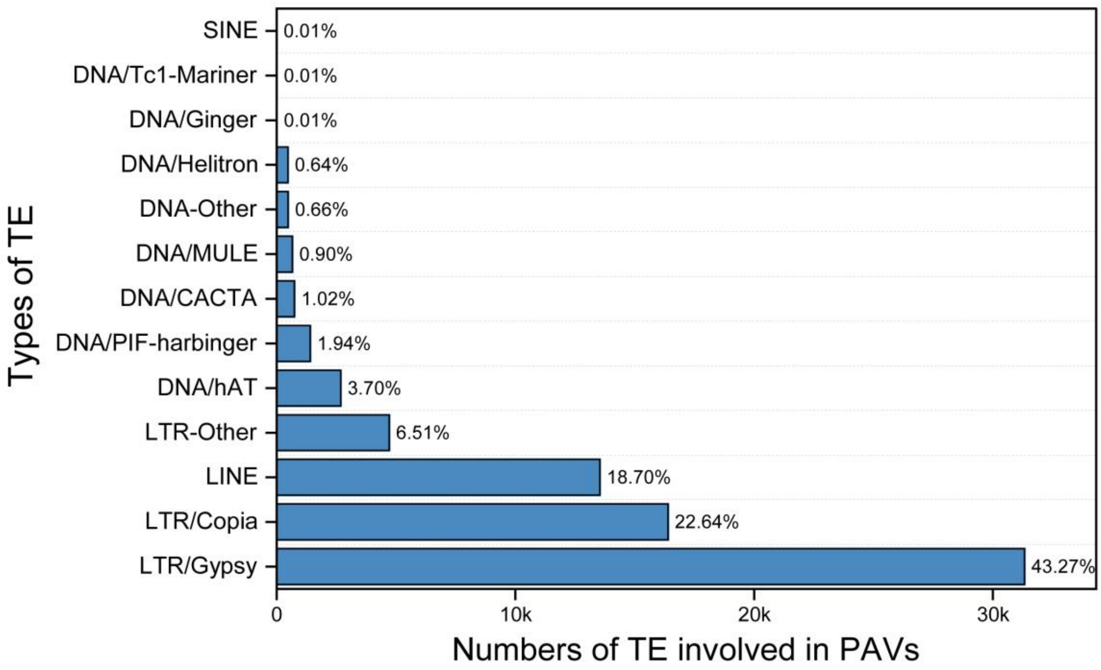

Supplementary Fig. 8. The distribution of TE types in PAVs.

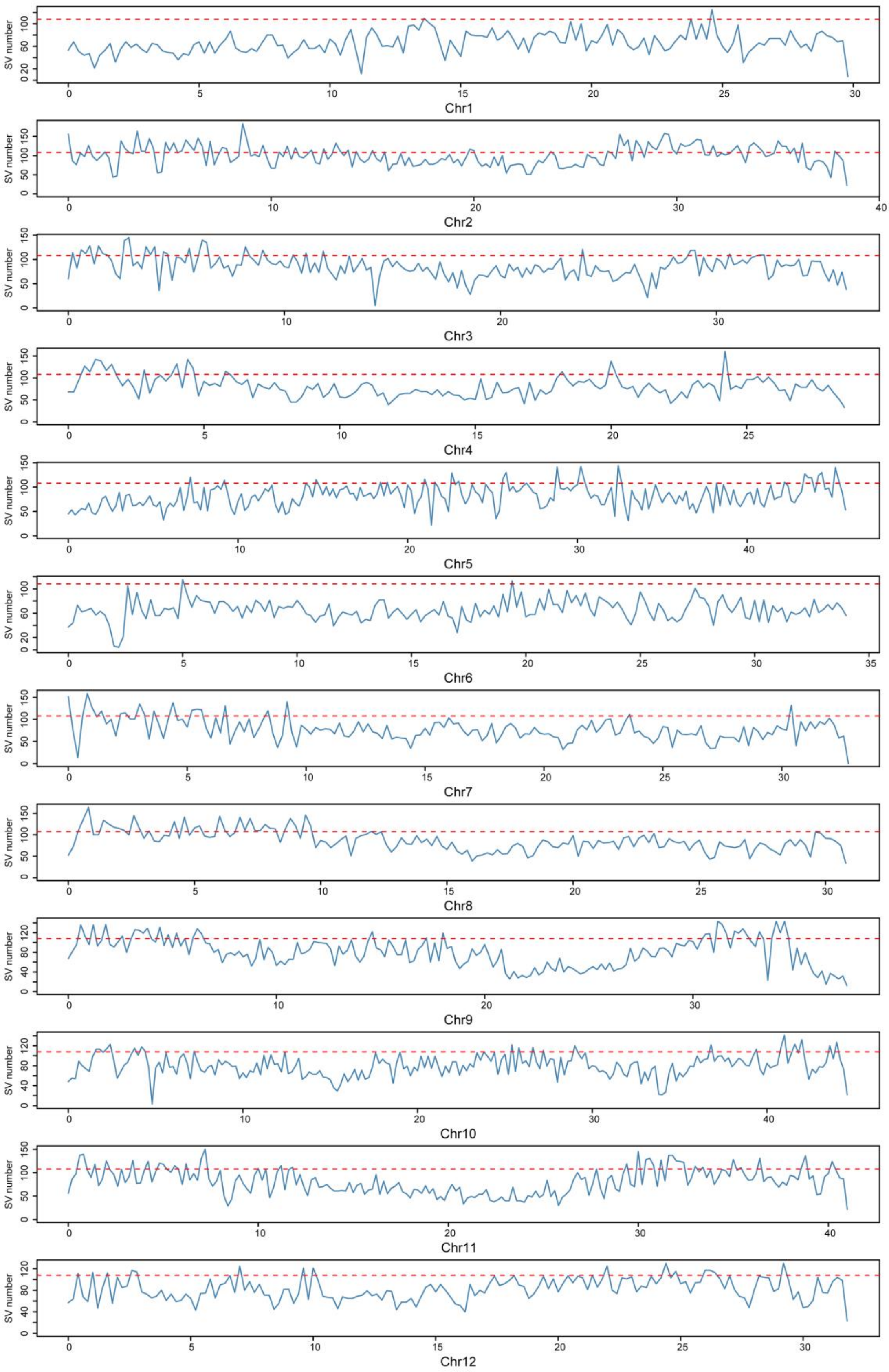

Supplementary Fig. 9. SV density distribution in the *Malus* pan-genome. The SV number in successive each 400 kb window (with a 200 kb step size) along 1 to 12 chromosome. The top 10% of all windows with the highest frequency of SV breakpoints as SV hotspots are shown on the figure.

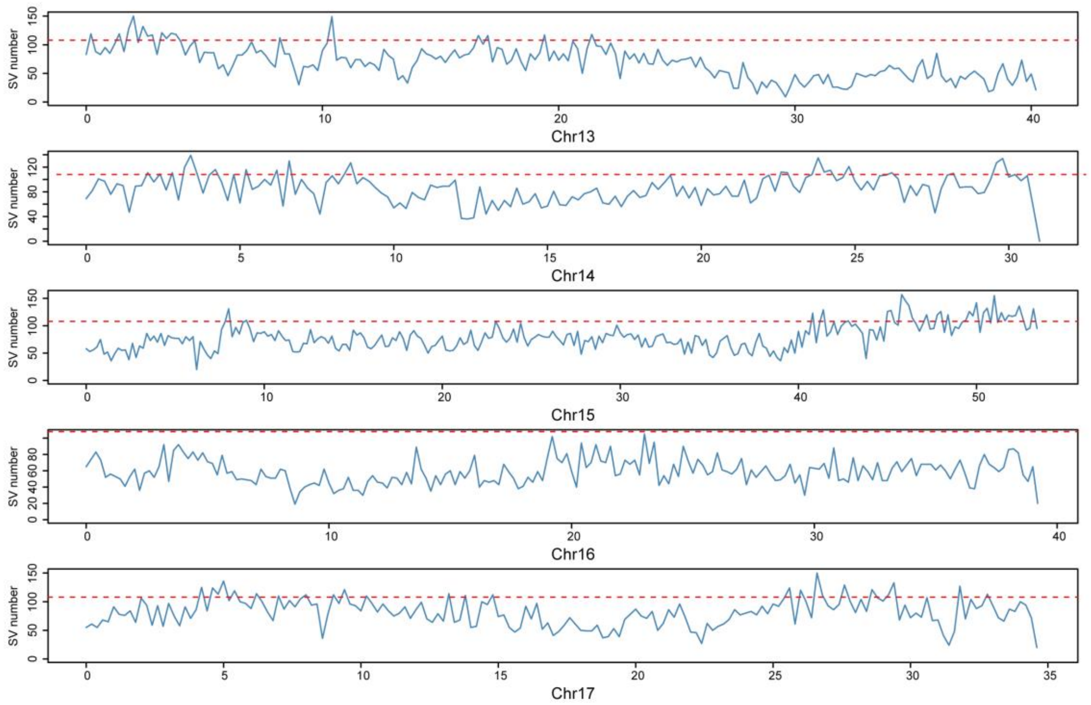

Supplementary Fig. 10. SV density distribution in the *Malus* pan-genome. The SV number in successive each 400 kb window (with a 200 kb step size) along 13-17 chromosome. The top 10% of all windows with the highest frequency of SV breakpoints as SV hotspots are shown on the figure.

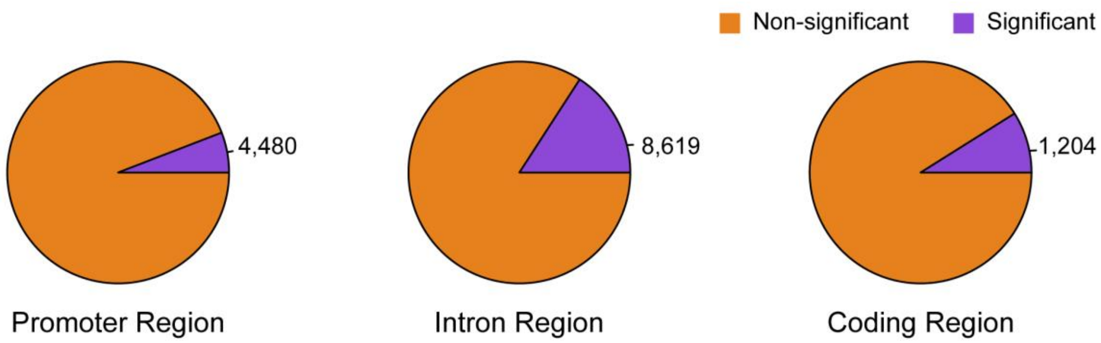

Supplementary Fig. 11. SVs overlapping the promoter, coding and intron regions significantly associated with altered expression of the corresponding genes in three different organs/tissues (leaves, fruit peels and fruit flesh). Significance was determined with Student's *t*-test at $p < 0.05$ (two-sided).

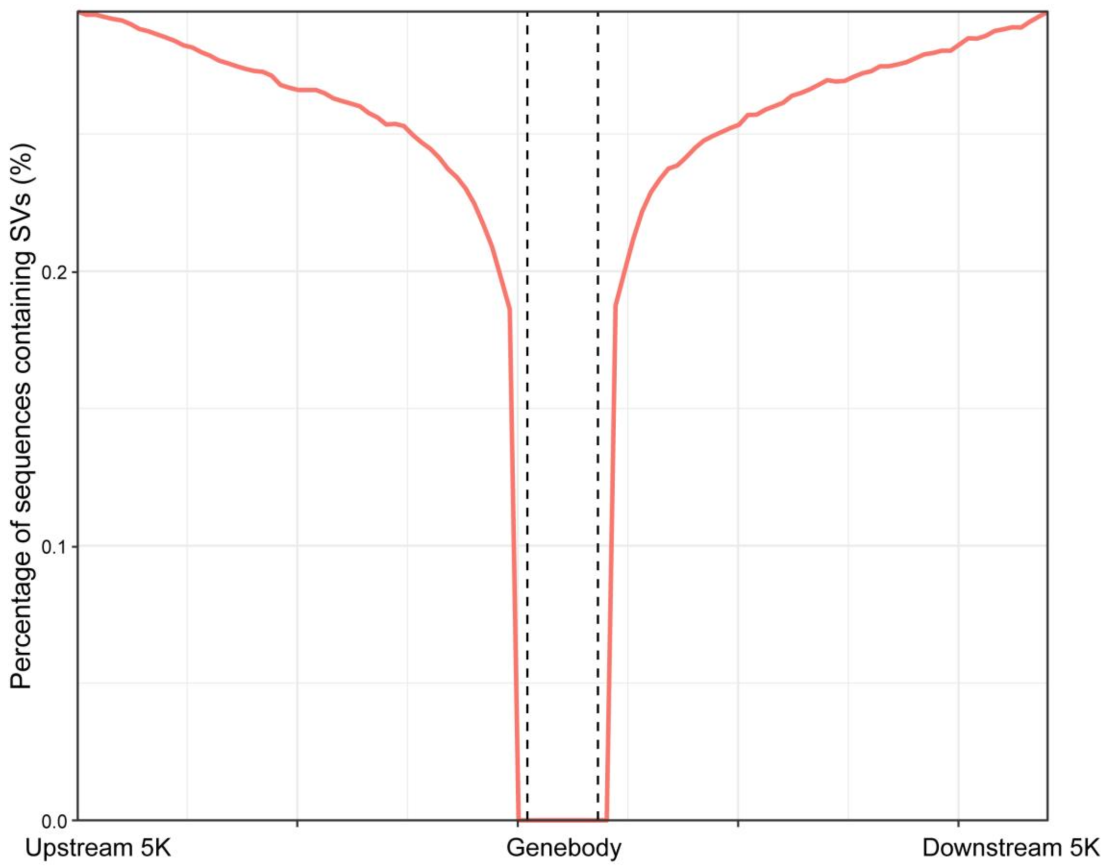

Supplementary Fig. 12. The distribution of SVs upstream and downstream from genes. Source data are provided as a Source Data file.

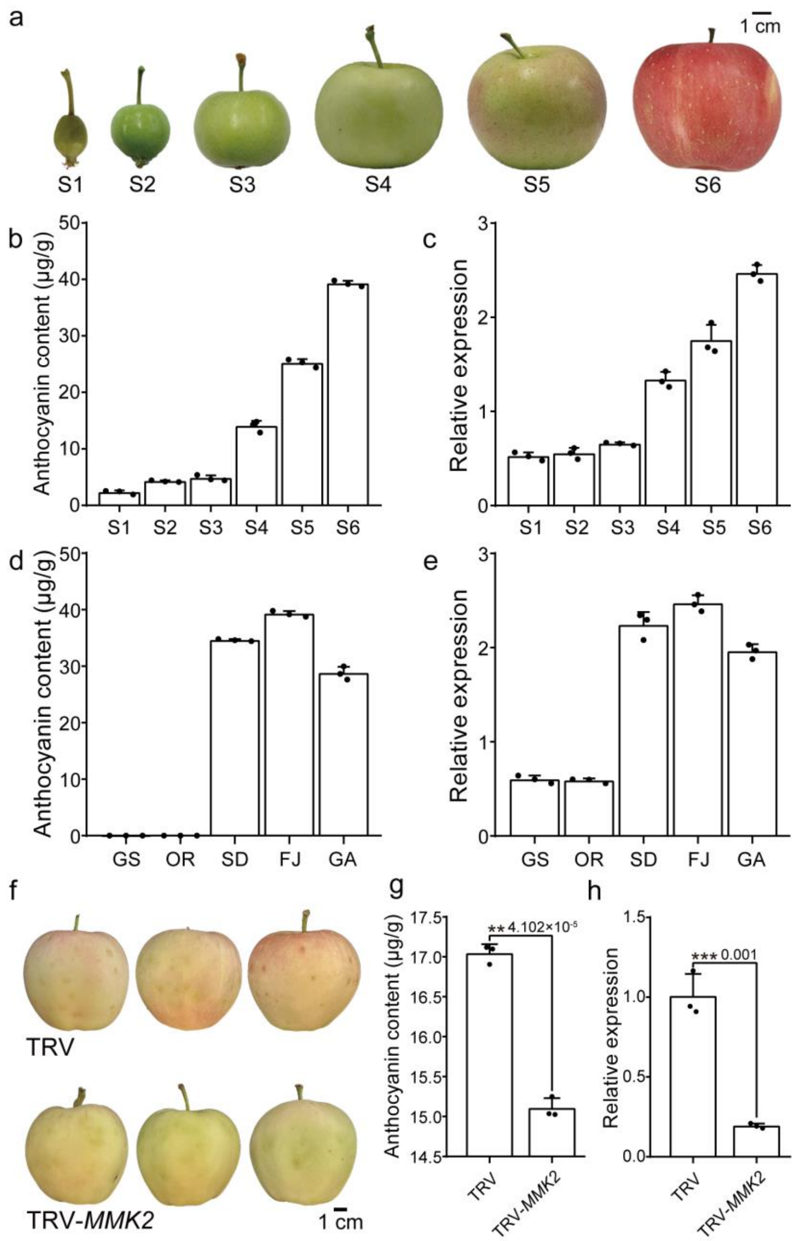

Supplementary Fig. 13. The correlation between *MdMMK2* expression and fruit color. a Fruit phenotypes of 'Red Fuji' during different developmental stages. Scale bar 1 cm. **b** Anthocyanin contents in apple peels during different 'Red Fuji' fruit developmental stages. The data are presented as mean values ± SD of three independent biological replicates). **c** Relative expression level of *MdMMK2* during different 'Red Fuji' fruit developmental stages. The data are presented as mean values ± SD of three independent biological replicates). **d** Anthocyanin contents in apple peels in different apple cultivars. The data are presented as mean values ± SD of three independent biological replicates). **e** Relative expression level of *MdMMK2* in apple peels in different apple cultivars. The data are presented as mean values ± SD three independent biological replicates). **f** Typical phenotypes of *MMK2* silenced (TRV-*MMK2*) 'Gala' fruit peels. Scale bar 1 cm. **g** Anthocyanin contents of *MMK2* silenced (TRV-*MMK2*) 'Gala' fruit peels. **h** Relative expression of *MMK2* in *MMK2* silenced (TRV-*MMK2*) 'Gala' fruit peels. Data are presented as mean values ± SD of three independent biological replicates. Asterisks indicate significant difference by Student's *t*-test (two-

sided; $^{**}p < 0.01$, $^{***}p < 0.001$), *p*-values are shown in **g** and **h**. Source data are provided as a Source Data file.

Supplementary Table 1. Hifi Summary of Hifi sequencing data.

Accession	GS	MO	HC	JG	COP	RA	OR	SD	MA	FJ
**High quality bases (Gb)**	37.74	25.91	19.75	28.27	29.24	31.43	20.62	20.86	21.45	25.76
**High quality reads**	2,701,970	1,835,352	1,424,675	1,901,624	2,386,553	1,812,037	1,222,660	1,615,777	1,409,875	1,835,165
**Average length (bp)**	13,966	14,115	13,866	14,864	12,250	17,343	16,861	12,910	15,211	14,035
**Max length (bp)**	39,635	67,033	62,072	70,862	66,326	69,969	69,455	64,110	59,700	44,687
**Reads N50* (bp)**	14,127	15,033	13,529	16,404	12,447	18,427	17,508	13,377	15,530	14,198
**Coverage**	53.15	36.49	27.82	39.81	41.18	44.26	29.04	29.38	30.21	36.28

Note: Coverage is calculated based on the estimated haploid genome size of 710 Mb[1]

Supplementary Table 2. Features of ten *Malus* genomic assemblies.

Genome	Estimated assembly size (Mb)	Estimated heterozygous_rate (%)	Estimated repeat_rate (%)	Total assembly size (bp)	Total contig size (bp)	Contig N50 (bp)	Busco	Cegma	LAI
**GS**	668.25	1.13	58.59	664,332,865	664,324,665	32,800,000	96.90%	97.58	20.01
**MO**	672.42	1.06	56.58	665,066,853	665,039,553	33,272,192	95.40%	96.37	19.47
**HC**	673.5	1.67	56.08	661,830,680	661,827,080	24,064,481	96.80%	97.58	18.44
**JG**	672.27	1.05	57.06	663,586,809	663,586,409	37,230,942	97.10%	97.58	18.02
**COP**	672.51	0.99	57.72	668,028,840	668,027,340	39,172,733	98.10%	96.77	19.18
**RA**	676.11	0.98	59.14	662,879,915	662,877,615	32,792,745	96.80%	96.77	18.88
**OR**	679.75	0.88	57.71	668,745,562	668,744,762	37,013,397	98.50%	96.37	18.1
**SD**	672.09	0.93	57.7	662,069,590	662,068,290	36,685,131	98.30%	97.58	19.18
**MA**	678.05	1.5	56.35	662,379,014	662,377,914	36,166,843	97.90%	97.98	20.6
**FJ**	670.44	0.99	57.64	665,442,561	665,439,161	32,991,989	98.00%	97.98	18.13

Supplementary Table 3. Evaluation of valid data for Hi-C sequences.

Accession	GS		FJ	
	Number of reads	Percentage %	Number of reads	Percentage %
**Total pairs processed**	408,273,432	100	333,357,177	100
**Unique paired alignments**	146,265,301	35.83	116,376,324	34.91
**Valid interaction pairs**	123,266,041	30.19	113,306,263	33.99
**Dangling end pairs**	2,857,914	0.7	2,260,397	0.68
**Religation pairs**	1,252,622	0.31	732,226	0.22
**Self cycle pairs**	1,309,428	0.32	70,304	0.02
**Dumped pairs**	3,238	<0.01	7,134	<0.01

Supplementary Table 4. Summary of Illumina Hi-C sequences.

Accession	GS	FJ
**Read pairs number**	408,273,432	333,357,177
**Base number (bp)**	122,482,029,600	100,007,153,100
**GC content (%)**	37.94	39.88
**Q20 ratio (%)**	97.6	96.71
**Q30 ratio (%)**	93.1	91.34
**Mapped ratio (%)**	97.74	97.18
**Unique mapped read pairs**	9,210,132	9,388,939

Supplementary Table 5. Completeness assessment of ten apple genomes using BUSCO (Benchmarking Universal Single-Copy Orthologs).

Accession	BUSCO assessment results
**GS**	C:96.9%[S:60.8%,D:36.1%],F:0.8%,M:2.3%,n:2326
**MO**	C:95.4%[S:58.5%,D:36.9%],F:0.9%,M:3.7%,n:2326
**HC**	C:96.8%[S:57.9%,D:38.9%],F:0.8%,M:2.4%,n:2326
**JG**	C:97.1%[S:57.4%,D:39.7%],F:0.7%,M:2.2%,n:2326
**COP**	C:98.1%[S:59.8%,D:38.3%],F:0.7%,M:1.2%,n:2326
**RA**	C:96.8%[S:58.0%,D:38.8%],F:0.6%,M:2.6%,n:2326
**OR**	C:98.5%[S:59.5%,D:39.0%],F:0.6%,M:0.9%,n:2326
**SD**	C:98.3%[S:59.6%,D:38.7%],F:0.6%,M:1.1%,n:2326
**MA**	C:97.9%[S:58.2%,D:39.7%],F:0.7%,M:1.4%,n:2326
**FJ**	C:98.0%[S:59.3%,D:38.7%],F:0.7%,M:1.3%,n:2326

Supplementary Table 6. Assessment of the LTR completeness using LAI.

Accession	Length of genome (bp)	Intact	Total	Raw_LAI	LAI
**GS**	664,332,865	0.0813	0.4699	17.56	20.01
**MO**	665,066,853	0.0879	0.5052	17.39	19.47
**HC**	661,830,680	0.0829	0.5046	16.44	18.44
**JG**	663,586,809	0.0873	0.5074	17.21	18.02
**COP**	668,028,840	0.0871	0.5027	17.33	19.18
**RA**	662,879,915	0.0873	0.5105	17.09	18.88
**OR**	668,745,562	0.0894	0.5105	17.52	18.1
**SD**	662,069,590	0.0884	0.5089	17.37	19.18
**MA**	662,379,014	0.0836	0.4954	16.88	20.6
**FJ**	665,442,561	0.0853	0.5034	16.95	18.13

Supplementary Table 7. Statistics for protein-coding gene prediction of ten *Malus* genomes.

Accession	GS	MO	HC	JG	COP	RA	OR	SD	MA	FJ
**Number of genes**	46,050	45,167	45,541	45,653	45,547	45,373	45,320	45,027	45,359	45,948
**Average mRNA length (CDS+intron)**	4,007.29	3,842.80	3,474.98	3,895.44	3,798.24	3,621.07	3,857.02	3,896.24	3,869.22	3,241.97
**Average CDS length**	1,131.55	1,097.94	1,103.70	1,116.80	1,118.21	1,105.08	1,127.73	1,133.11	1,125.90	1,075.71
**Average exon length**	231.55	225.37	229.6	226.39	226.89	228.79	226.96	226.65	226.71	235.06
**Average exon number per gene**	4.89	4.87	4.81	4.93	4.93	4.83	4.97	5	4.97	4.58
**Average intron length**	739.86	708.96	622.87	706.47	682.21	656.89	687.67	690.89	691.65	605.72

Supplementary Table 8. Summary of RNA-sequencing data.

Sample	Raw reads	Clean reads	Raw base (G)	Clean base (G)	Effective rate (%)	Error rate (%)	Q20 (%)	Q30 (%)	GC content (%)
**GS-1**	22,748,437	21,878,680	6.82	6.56	96.18	0.03	97.72	93.5	46.11
**GS-2**	23,197,181	22,285,380	6.96	6.69	96.07	0.03	97.05	91.9	46.97
**GS-3**	22,949,659	22,534,496	6.88	6.76	98.19	0.03	97.69	93.44	47.4
**MO-1**	20,383,691	19,315,971	6.12	5.79	94.76	0.03	97.81	93.89	47.29
**HC-1**	22,900,774	22,065,791	6.87	6.62	96.35	0.03	97.5	93	46.84
**MSM-1**	21,812,166	20,859,416	6.54	6.26	95.63	0.03	97.66	93.41	46.55
**JG-1**	22,798,375	21,815,115	6.84	6.54	95.69	0.03	97.94	94.13	47.09
**COP-1**	20,300,959	19,637,658	6.09	5.89	96.73	0.03	97.57	93.22	47.3
**RA-1**	22,432,681	21,047,867	6.73	6.31	93.83	0.03	97.71	93.58	46.55
**MSR-1**	21,755,057	21,210,162	6.53	6.36	97.5	0.03	97.54	93.16	45.74
**OR-1**	20,184,354	19,393,121	6.06	5.82	96.08	0.03	97.9	94.09	46.75
**OR-2**	21,402,929	20,474,682	6.42	6.14	95.66	0.03	97.81	93.96	48.63
**OR-3**	23,344,169	22,590,783	7	6.78	96.77	0.03	97.91	94.03	47.11
**GA-1**	21,569,911	20,847,895	6.47	6.25	96.65	0.03	97.72	93.55	46.01
**GA-2**	21,753,549	21,122,055	6.53	6.34	97.1	0.03	97.38	92.87	45.66
**GA-3**	21,893,803	21,296,660	6.57	6.39	97.27	0.03	97	92.31	45.17
**SD-1**	22,189,699	21,469,086	6.66	6.44	96.75	0.03	97.53	93.12	46.53
**SD-2**	23,048,361	22,240,152	6.91	6.67	96.49	0.03	97.68	93.41	46.66
**SD-3**	22,422,454	22,094,815	6.73	6.63	98.54	0.03	97.31	92.65	46.33
**MA-1**	20,471,615	19,164,404	6.14	5.75	93.61	0.03	97.62	93.56	46.63
**FJ-1**	22,285,680	21,431,990	6.69	6.43	96.17	0.03	97.59	93.24	47.07
**FJ-3**	21,364,933	21,043,759	6.41	6.31	98.5	0.03	97.75	93.59	47.35

Note: 1: leaves; 2: fruit peel; 3: fruit flesh

Supplementary Table 9. Gene clustering result of *Malus* genomes.

Sample	Gene number	Cluster number	Core cluster	Core number	Soft-core cluster	Soft-core number	Accessory (dispensable) cluster	Accessory (Dispensable) number	Specific cluster	Specific number
**COP**	45,547	33,619	14,896	21,843	7,314	9,891	11,381	13,785	1,423	1,448
**FJ**	45,948	32,683	14,896	21,850	7,068	9,566	10,685	14,498	2,608	2,720
**GA**	45,352	30,037	14,896	22,909	6,314	9,063	8,667	13,220	823	1,078
**GS**	46,050	33,182	14,896	21,375	7,141	9,642	11,114	15,002	1,202	2,196
**HC**	45,541	32,412	14,896	22,130	6,866	9,250	10,598	14,109	2,381	2,793
**JG**	45,653	32,973	14,896	22,158	7,101	9,674	12,762	15,607	2,807	2,837
**MO**	45,167	32,960	14,896	22,186	6,708	9,396	11,044	13,273	995	1,027
**MSM**	45,199	29,256	14,896	23,266	6,533	9,407	8,226	12,925	1,377	1,671
**MSR**	45,210	30,358	14,896	22,528	6,411	9,089	8,867	13,409	833	1,029
**OR**	45,320	33,040	14,896	21,924	7,408	9,930	10,653	13,383	833	859
**RA**	45,373	32,099	14,896	21,716	7,039	9,094	9,706	14,105	904	955
**SD**	45,027	32,885	14,896	21,739	7,400	9,807	11,132	14,024	1,306	1,315
**MA**	45,359	32,841	14,896	22,244	7,248	9,950	10,349	12,817	766	932

Supplementary Table 10. Annotation of identified CNVs in 12 *Malus* genomes.

CNV	MO	HC	MSM	JG	COP	RA	MSR	OR	GA	SD	MA	FJ
**Delection**	252	532	448	290	275	418	314	744	225	438	517	492
**Duplication**	767	430	877	719	775	554	372	748	368	903	560	989
**Downstream**	5	1	10	2	6	4	6	6	6	5	3	12
**Exonic**	869	772	980	860	857	798	429	1,226	428	1,141	845	1,150
**Intergenic**	134	180	324	145	180	164	244	247	156	182	219	302
**Intronic**	3	2	2	2	4	4	1	5	0	9	5	6
**Upstream**	8	7	9	0	3	2	6	8	3	4	5	11

Supplementary Table 11. Summary of identified variations in 12 *Malus* genomes.

Accession	Types	Inversions	Translocations	Presence	Absence
	**Count**	139	8,947	34,373	34,024
**MO**	**Length_ref**	25,494,385	39,252,790	--	137,418,039
	**Length_qry**	27,537,991	35,994,728	151,650,734	--
	**Count**	210	14,005	50,592	50,941
**HC**	**Length_ref**	18,452,094	37,669,587	--	164,444,289
	**Length_qry**	18,871,046	37,834,290	180,951,848	--
	**Count**	178	10,241	41,658	42,255
**MSM**	**Length_ref**	20,512,169	28,088,906	--	140,846,242
	**Length_qry**	19,593,264	28,075,899	144,338,588	--
	**Count**	146	8,060	33,835	33,602
**JG**	**Length_ref**	23,027,977	22,230,802	--	122,158,971
	**Length_qry**	28,189,056	22,097,895	139,790,435	--
	**Count**	145	8,310	33,718	33,664
**COP**	**Length_ref**	18,241,693	25,787,685	--	112,024,759
	**Length_qry**	21,109,045	26,398,883	139,115,141	--
	**Count**	131	8,150	33,387	33,494
**RA**	**Length_ref**	17,964,701	25,789,150	--	114,459,562
	**Length_qry**	20,127,214	25,402,341	131,344,929	--
	**Count**	172	9,875	38,679	40,372
**MSR**	**Length_ref**	22,225,995	24,620,213	--	134,158,982
	**Length_qry**	26,396,966	24,874,228	138,027,104	--
	**Count**	135	7,833	33,073	33,185
**OR**	**Length_ref**	24,223,557	24,182,540	--	108,035,540
	**Length_qry**	27,184,062	23,968,384	135,992,353	--
	**Count**	179	7,591	30,273	34,145
**GA**	**Length_ref**	23,933,928	23,104,305	--	123,747,141
	**Length_qry**	24,999,789	22,829,837	105,605,690	--
	**Count**	143	8,892	35,859	35,586
**SD**	**Length_ref**	19,023,344	21,900,975	--	114,367,251
	**Length_qry**	21,428,166	21,883,549	137,493,134	--
	**Count**	159	10,873	42,886	43,798
**MA**	**Length_ref**	23,796,816	29,430,540	--	142,874,405
	**Length_qry**	24,913,610	29,478,504	148,844,597	--
	**Count**	134	8,241	33,893	34,136
**FJ**	**Length_ref**	25,653,241	24,397,669	--	119,862,337
	**Length_qry**	30,573,076	24,083,853	130,950,509	--

Supplementary Table 12. SV annotation statistics.

Insertion	Deletion	Upstream	Frameshift deletion	Frameshift insertion	Nonframeshift deletion	Nonframeshift insertion	Intronic	Splicing	Downstream	Upstream/Downstream	Intergenic
152,482	98,080	46,153	6,903	2,727	1,950	1,406	54,385	131	37,810	30,342	68,243

Note: “Upstream/Downstream” represents SNPs which located between two genes (upstream of one gene and downstream of another gene).

Supplementary Table 13. List of genotype identification of 24 cultivars.

Name	Taxonomy	Origin	Skin color
Spokane Beauty	*Malus domestica* (Suckow) Borkh.	Washington, United States	70% carmine stripe, slight russet
Jonafree	*Malus domestica* (Suckow) Borkh.	Illinois, United States	75% medium red with smooth russet-free
Redfree	*Malus domestica* (Suckow) Borkh.	Indiana, United States	90% bright red
Smith Jonathan	*Malus domestica* (Suckow) Borkh.	Unknown	100% dark red
Jonsib Crab	*Malus* hybr.	South Dakota, United States	Brilliant red
Haralson	*Malus domestica* (Suckow) Borkh.	Minnesota, United States	Red-striped to deep red
Liberty	*Malus domestica* (Suckow) Borkh.	Washington, United States	Yellow blushed and striped red
Ingol	*Malus domestica* (Suckow) Borkh.	Germany	80% red
Viking	*Malus domestica* (Suckow) Borkh.	Wisconsin, United States	80-100% dark purplish red
Sweet Delicious	*Malus domestica* (Suckow) Borkh.	New York, United States	90% red
Mollie's Delicious	*Malus domestica* (Suckow) Borkh.	New Jersey, United States	70-90% red
Marshall McIntosh	*Malus domestica* (Suckow) Borkh.	Massachusetts, United States	More red color than McIntosh
Prima	*Malus* hybr.	Illinois, United States	60-95% red
Redspur Delicious	*Malus domestica* (Suckow) Borkh.	Washington, United States	Indistinguishable from Starking Delicious, except redder.
Burgundy	*Malus domestica* (Suckow) Borkh.	New York, United States	blackish, red fruit with solid blush, but without stripes
Murray	*Malus domestica* (Suckow) Borkh.	Ontario, Canada	red blush-stripe on green to yellow ground
Northern Spy	*Malus domestica* (Suckow) Borkh.	United States	greenish yellow blushed pinkish red with occasional russet patches
Monroe	*Malus domestica* (Suckow) Borkh.	New York, United States	pale greenish yellow
Rhode Island Greening	*Malus domestica* (Suckow) Borkh.	Unknown	yellowish green with occasional orange flush
Reinette Simirenko	*Malus domestica* (Suckow) Borkh.	Former, Soviet Union	yellowish green with a faint brownish orange blush
Dorsett Golden	*Malus domestica* (Suckow) Borkh.	Washington, United States	golden yellow
Winter Majetin	*Malus domestica* (Suckow) Borkh.	United Kingdom	greenish-yellow, red-brown blush
Calville Blanc	*Malus domestica* (Suckow) Borkh.	Belgium	yellow with light red flush
Sumatovka	*Malus domestica* (Suckow) Borkh.	Unknown	70-100% red, striped, attractive

Note: *Malus* species in this list with taxonomy, habitat and skin color information from the USDA GRIN database.

Supplementary reference

1. Zhang, L. et al. A high-quality apple genome assembly reveals the association of a retrotransposon and red fruit colour. *Nat. Commun*. **10**, 1494 (2019).
