# Supplementary material for: Pan-genome analysis of 13 Malus accessions reveals structural and sequence variations associated with fruit traits
Source: Nat Commun. 2023 Nov 15;14:7377. doi: 10.1038/s41467-023-43270-7 (PMC10651928; doi:10.1038/s41467-023-43270-7)
Supplement: Supplementary file 3 — Description of Additional Supplementary Files [file 41467_2023_43270_MOESM3_ESM.pdf]

Description of Additional Supplementary Files

File Name: Supplementary Data 1
Description: Summary of the transposon elements (TEs) for ten *Malus* genomes.

File Name: Supplementary Data 2
Description: Overview of functional annotation of the ten *Malus* genomes.

File Name: Supplementary Data 3
Description: KEGG of the expanded genes in FJ, RA and JG.

File Name: Supplementary Data 4
Description: Genes identified under positive selection in MO and their functional annotation.

File Name: Supplementary Data 5
Description: Genes identified under positive selection in SD and their functional annotation.

File Name: Supplementary Data 6
Description: Genes identified under positive selection in COP and their functional annotation.

File Name: Supplementary Data 7
Description: Genes identified under positive selection in FJ and their functional annotation.

File Name: Supplementary Data 8
Description: Genes identified under positive selection in GS and their functional annotation.

File Name: Supplementary Data 9
Description: Genes identified under positive selection in HC and their functional annotation.

File Name: Supplementary Data 10
Description: Genes identified under positive selection in JG and their functional annotation.

File Name: Supplementary Data 11
Description: Genes identified under positive selection in OR and their functional annotation.

File Name: Supplementary Data 12
Description: Genes identified under positive selection in RA and their functional annotation.

File Name: Supplementary Data 13
Description: Genes identified under positive selection in MA and their functional annotation.

File Name: Supplementary Data 14
Description: The number of gCNVs in *Malus* assemblies.

File Name: Supplementary Data 15
Description: The function annotation of gCNVs in 13 *Malus* fruit peels.

File Name: Supplementary Data 16
Description: The function annotation of gCNVs in 13 *Malus* fruit flesh.

File Name: Supplementary Data 17
Description: The function annotation of gCNVs in 13 *Malus* leaves.

File Name: Supplementary Data 18
Description: Enrichment analysis of SV hotspot region genes.

File Name: Supplementary Data 19
Description: KEGG of SV hotspot region genes on chromosome 8.

File Name: Supplementary Data 20
Description: KEGG of SV hotspot region genes on chromosome 13.

File Name: Supplementary Data 21
Description: Shared SVs associated with fruit color and biotic resistance.

File Name: Supplementary Data 22
Description: Primer sequences used in this study.
