# Supplementary material for: Pan-genome analysis of 13 Malus accessions reveals structural and sequence variations associated with fruit traits
Source: Nat Commun. 2023 Nov 15;14:7377. doi: 10.1038/s41467-023-43270-7 (PMC10651928; doi:10.1038/s41467-023-43270-7)
Supplement: Supplementary file 26 — Reporting Summary [file 41467_2023_43270_MOESM26_ESM.pdf]

nature portfolio

Corresponding author(s): Ting Wu, Zhenhai Han

Last updated by author(s): Oct 17, 2023

Reporting Summary

Nature Portfolio wishes to improve the reproducibility of the work that we publish. This form provides structure for consistency and transparency in reporting. For further information on Nature Portfolio policies, see our Editorial Policies and the Editorial Policy Checklist.

Statistics

For all statistical analyses, confirm that the following items are present in the figure legend, table legend, main text, or Methods section.

n/a	Confirmed	
☐	☒	The exact sample size (*n*) for each experimental group/condition, given as a discrete number and unit of measurement
☐	☒	A statement on whether measurements were taken from distinct samples or whether the same sample was measured repeatedly
☐	☒	The statistical test(s) used AND whether they are one- or two-sided<br>*Only common tests should be described solely by name; describe more complex techniques in the Methods section.*
☒	☐	A description of all covariates tested
☐	☒	A description of any assumptions or corrections, such as tests of normality and adjustment for multiple comparisons
☐	☒	A full description of the statistical parameters including central tendency (e.g. means) or other basic estimates (e.g. regression coefficient) AND variation (e.g. standard deviation) or associated estimates of uncertainty (e.g. confidence intervals)
☐	☒	For null hypothesis testing, the test statistic (e.g. *F*, *t*, *r*) with confidence intervals, effect sizes, degrees of freedom and *P* value noted<br>*Give P values as exact values whenever suitable.*
☒	☐	For Bayesian analysis, information on the choice of priors and Markov chain Monte Carlo settings
☒	☐	For hierarchical and complex designs, identification of the appropriate level for tests and full reporting of outcomes
☒	☐	Estimates of effect sizes (e.g. Cohen's *d*, Pearson's *r*), indicating how they were calculated

Our web collection on statistics for biologists contains articles on many of the points above.

Software and code

Policy information about availability of computer code

Data collection	No commercial code was used in this study.
Data analysis	All of the software and code used in this study can be found in the Methods section. ccs software (v3.0.0); Jellyfish (v2.2.6); HiC-Pro (v2.11.1); LACHESIS (v2.0); Juicebox (v1.11.08); NUCmer (v3.23); BUSCO (v2); MCScanX (v1) LAI retriever (v2.8.7); LTR_FINDER (v1.0.5); PILER; WUblast (v2.0); Solar software (v1.0); GeneWisev (v2.4.1); PASA (v2.3.3); Augustus (v2.5.5); Genscan (v1.0); Geneid (v1.4); GlimmerHMM (v3.0.1); SNAP (2013.11.29); Tophat (v2.0.8); Cufflinks (v2.1.1); EVidenceModeler (v1.1.1); OrthoFinder (v1.1.4); Muscle (v3.8.31); RAxML (v8.0.19); IQ-TREE (v1.6.6); PAML (v4.8); CAFÉ software (v2.1); NUCmer (v3.23); SyRI□20-06-2022□; Minimap2 (v2.24); CNVnator (v0.4.1); BLAT (v36); Hisat2 (v2.0.5); HTSeq (v0.6.1); DESeq2 R package (v1.20.0); GraphPad Prism 8.0; IBM SPSS Statistics 22.

For manuscripts utilizing custom algorithms or software that are central to the research but not yet described in published literature, software must be made available to editors and reviewers. We strongly encourage code deposition in a community repository (e.g. GitHub). See the Nature Portfolio guidelines for submitting code & software for further information.

Data

Policy information about availability of data

All manuscripts must include a data availability statement. This statement should provide the following information, where applicable:

- Accession codes, unique identifiers, or web links for publicly available datasets
- A description of any restrictions on data availability
- For clinical datasets or third party data, please ensure that the statement adheres to our policy

The raw sequencing data have been deposited in the National Genomics Data Center BioProject no. PRJNA872768 (https://www.ncbi.nlm.nih.gov/search/all/?term=PRJNA872768). The genome sequences are accessible under NCBI BioProject no. PRJNA869488 (https://www.ncbi.nlm.nih.gov/search/all/?term=PRJNA869488) and no. PRJNA927238 (https://www.ncbi.nlm.nih.gov/search/all/?term=PRJNA927238). The related cluster data have now been uploaded to figshare and have the download link is: https://figshare.com/s/4e1ea61459393ff54684. The related PAV data were uploaded to figshare and the download link is https://figshare.com/s/c6734dc864c07f51df9c. The related GFF files were uploaded to figshare and the download link is: https://figshare.com/s/5fea32dd0fba11ead6bd.

Research involving human participants, their data, or biological material

Policy information about studies with human participants or human data. See also policy information about sex, gender (identity/presentation), and sexual orientation and race, ethnicity and racism.

Reporting on sex and gender	N/A
Reporting on race, ethnicity, or other socially relevant groupings	N/A
Population characteristics	N/A
Recruitment	N/A
Ethics oversight	N/A

Note that full information on the approval of the study protocol must also be provided in the manuscript.

Field-specific reporting

Please select the one below that is the best fit for your research. If you are not sure, read the appropriate sections before making your selection.

☒ Life sciences ☐ Behavioural & social sciences ☐ Ecological, evolutionary & environmental sciences

For a reference copy of the document with all sections, see nature.com/documents/nr-reporting-summary-flat.pdf

Life sciences study design

All studies must disclose on these points even when the disclosure is negative.

Sample size	We assembled 10 high-quality genomes of genetically diverse Malus accessions. Three biological replicates of the fruit and calli samples were analyzed. Experiments (RT-qPCR and Anthocyanin content) were repeated three times independently with similar results. The Dual-LUC activity assays experiments are independently performed six biological replicates. For comparison of two groups, we used Student's t-test (two-sided; * p < 0.05, ** p < 0.01, *** p < 0.001). For multiple comparisons, ANOVA was used followed by Duncan's test (p < 0.05). All attempts at replications were successful.
Data exclusions	No data was excluded for the analysis.
Replication	Three biological replicates of the fruit and calli samples were analyzed. Two biological replicates of RNAseq samples were performed.
Randomization	The plants were randomly allocated in glasshouse and field.
Blinding	This is not relevant for the method. Blinding is not applicable in our study because it does not involve subjects which receive different treatments. All experiments were done by analyzing data derived from different biological replicates directly.

Reporting for specific materials, systems and methods

We require information from authors about some types of materials, experimental systems and methods used in many studies. Here, indicate whether each material, system or method listed is relevant to your study. If you are not sure if a list item applies to your research, read the appropriate section before selecting a response.

Materials & experimental systems

n/a	Involved in the study	
☒	☐	Antibodies
☒	☐	Eukaryotic cell lines
☒	☐	Palaeontology and archaeology
☒	☐	Animals and other organisms
☒	☐	Clinical data
☒	☐	Dual use research of concern
☐	☒	Plants

Methods

n/a	Involved in the study	
☒	☐	ChIP-seq
☒	☐	Flow cytometry
☒	☐	MRI-based neuroimaging

Dual use research of concern

Policy information about dual use research of concern

Hazards

Could the accidental, deliberate or reckless misuse of agents or technologies generated in the work, or the application of information presented in the manuscript, pose a threat to:

No	Yes	
☒	☐	Public health
☒	☐	National security
☒	☐	Crops and/or livestock
☒	☐	Ecosystems
☒	☐	Any other significant area

Experiments of concern

Does the work involve any of these experiments of concern:

No	Yes	
☒	☐	Demonstrate how to render a vaccine ineffective
☒	☐	Confer resistance to therapeutically useful antibiotics or antiviral agents
☒	☐	Enhance the virulence of a pathogen or render a nonpathogen virulent
☒	☐	Increase transmissibility of a pathogen
☒	☐	Alter the host range of a pathogen
☒	☐	Enable evasion of diagnostic/detection modalities
☒	☐	Enable the weaponization of a biological agent or toxin
☒	☐	Any other potentially harmful combination of experiments and agents
